# From Bronchodilation to Lactic Acidosis: A Case Report on Salbutamol’s Adverse Effect

**DOI:** 10.7759/cureus.63213

**Published:** 2024-06-26

**Authors:** Teresa Miranda, Marta Maio Herculano, João Sousa Torres, Francisco Das Neves Coelho, Marta Rebelo

**Affiliations:** 1 Intensive Care Department, Centro Hospitalar Lisboa Ocidental, Lisbon, PRT; 2 Helicopter Emergency Medical Service, Instituto Nacional de Emergencia Medica, Lisbon, PRT; 3 Intensive Care Department, Hospital Egas Moniz, Centro Hospitalar Lisboa Ocidental, Lisbon, PRT

**Keywords:** hyperlactacidemia, intensive care, asthma, lactic acidosis, short-acting beta-2 agonists

## Abstract

Asthma is one of the most prevalent chronic respiratory diseases, characterized by bronchial hyper-responsiveness and intermittent airflow obstruction. Short-acting β_2_ agonists (SABA) remain the cornerstone of acute asthma management due to its properties in smooth muscle relaxation and bronchodilatation. Rarely, these drugs might be associated with adverse effects, including the development of metabolic and hydro-electrolytic imbalances.

We report a case of lactic acidosis secondary to β_2_ agonists in a young female patient admitted with severe acute asthma. After initial management and significant improvement of the respiratory distress with nebulized and subcutaneous salbutamol, the patient developed high anion gap metabolic acidosis due to hyperlactacidemia and hypokalemia. Alternative causes of lactic acidosis were discarded, such as severe hypoxemia, systemic hypoperfusion, sepsis, and organ dysfunction, and SABA-related lactic acidosis was suspected. This treatment was halted, and tachypnea, metabolic acidosis, and lactate levels rapidly resolved. The remainder of the hospital stay was uneventful, and the patient was discharged after a period of five days. Although rare, the development of unexplained lactic acidosis in a SABA-treated patient should alert the treating physician to this β2 agonist side-effect.

## Introduction

Asthma is one of the most prevalent chronic respiratory diseases worldwide, and the cornerstone of its pathophysiology is intermittent airflow obstruction secondary to bronchial inflammation and hyperresponsiveness. Short-acting β2 agonists (SABA), including salbutamol, in combination with ipratropium bromide and oral/intravenous corticosteroids, remain one of the most used bronchodilator agents for acute asthma treatment. Nevertheless, this pharmacological class can be responsible for adverse effects, including lactic acidosis [1,2].

Lactic acidosis can mimic or exacerbate asthma symptoms, such as tachypnea, that can worsen the clinical status, potentially leading to mismanagement if not promptly identified. In cases of acute severe asthma, elevated blood lactate levels can also and most frequently arise from several factors: hypoxemia, inadequate tissue oxygen delivery due to hypotension, and overproduction by respiratory muscles due to increased workload. The timely recognition and adequate etiological management of lactic acidosis are fundamental for optimal outcomes [1,3]. We report a case of lactic acidosis in a patient with asthma exacerbation in the absence of tissue hypoperfusion and highlight the difficulties in the management and treatment of acute asthma exacerbations in this setting.

## Case presentation

A 23-year-old female patient, with a history of asthma; medication of inhaled formoterol/budesonide, prednisolone 60 mg/day, montelukast 10 mg/day, and omalizumab 300 mg subcutaneous every two weeks; poor therapy compliance; and without other relevant past history, was brought to the emergency department (ED) due to dyspnea and prostration. Upon admission, she was lethargic, with significant tachypnea. The patient was hemodynamically stable and dispersed bilateral wheezing was present in lung auscultation. The admission blood gas analysis (venturi mask with 60% fraction of inspired oxygen (FiO2)) showed pH 7.56, partial pressure of carbon dioxide (PaCO2) 20 mmHg, partial pressure of oxygen (PaO2) 140 mmHg, and lactate 0.4mmol/L. Laboratory workup was unremarkable, and chest X-ray was normal.

The patient was medicated with inhaled bronchodilators, hydrocortisone 200 mg, and magnesium sulfate 2 g and was later started on methylprednisolone and inhaled salbutamol/ipratropium bromide. Due to further decompensation, additional nebulized and subcutaneous salbutamol was administered, and she was transferred to the ICU with a diagnosis of severe acute asthma decompensation.

Upon ICU admission, a metabolic acidemia with increased anion gap due to hyperlactacidemia was recognized (arterial blood gas: pH 7.29, PaCO2 21.2 mmHg, PaO2 152 mmHg, bicarbonate (HCO3) 13.6 mmol/L, anion gap (AG) 20, hemoglobin (Hb) 12.4 g/dL, lactate 10.8 mmol/L) along with severe hypokalemia (K+ 2.2mmol/L). Other causes of hyperlactacidemia were thoroughly searched, including systemic hypoperfusion, sepsis, and organ dysfunction, leaving behind a diagnosis of lactic acidosis secondary to β2 agonists. Salbutamol was halted, and the tachypnea began to resolve. After four hours of discontinuation, lactate levels were normal and metabolic acidosis was compensated (Table 1). After two days in the ICU, the patient improved clinically and thereafter was transferred to the pneumology ward. After discharge, the patient stopped all her medication despite medical advice.

**Table 1:** Blood gas analysis evolution from the time of the patient's admission to ICU discharge

Parameters	On ED admission	On ICU admission	7 hours after ICU admission	Salbutamol stopped	9 hours after discontinuation of salbutamol	On ICU discharge
pH	7.56	7.41	7.29		7.47	7.43
PaCO2 (mmHg)	20	28.4	21.1		30.1	33.6
PaO2 (mmHg)	140	199	152		108	72.7
HCO3- (mmol/L)	22	20	13.6		21.5	22.5
AG	13	16.1	20		12	13
Lactate (mmol/L)	0.4	6.9	9.7		0.8	1

After five months, the patient was readmitted to the ICU due to another episode of severe asthma exacerbation, this time requiring invasive mechanical ventilation. During the ICU stay, the patient developed recurrent hyperlactatemia with metabolic acidosis, again due to SABA therapy, rapidly normalizing after the withdrawal of β2 agonists. To date, she maintains irregular follow-up and poor therapy compliance.

## Discussion

We report a case of lactic acidosis secondary to salbutamol in a patient presenting with severe asthma exacerbation. The awareness of this rare adverse effect and underlying mechanisms is of utmost importance to selecting appropriate management.

Salbutamol is a SABA and is used as a first-line drug to relieve acute airflow obstruction and symptoms in asthma, chronic obstructive pulmonary disease (COPD), and other bronchospasm reactions [4]. Its therapeutic effect is based on its potent smooth muscle relaxant properties, which generate bronchodilatation. Salbutamol has several routes of administration (oral, intravenously, intramuscularly, subcutaneously, inhalation). The inhaled form (spray or nebulization) has been considered efficient and safe, reducing the exposure of its systemic adverse effects.

Extrapulmonary β2-adrenergic receptor activation is responsible for SABA adverse effects such as cardiac rhythm disturbances, hypokalemia, and hyperlactacidemia. The cardiac rhythm disturbances result from the positive inotropic and chronotropic effects [5]. The effects on the potassium arise from the stimulation of the sodium (Na)+/potassium (K+)-adenosine triphosphatase (ATP) pump in skeletal muscle, which shifts K+ intracellularly, causing hypokalaemia [6]. The increased lactate production is the product of a diverted metabolism of the pyruvate from the Krebs cycle to lactate formation as a result of the activation of β2-stimulation and activation of glycolysis [7].

As a product of anaerobic glucose metabolism from pyruvate, lactate blood levels above 2 mmol/L may evolve into lactic acidosis, classified as type A (ischemic origin) or type B (other sources). In both types, mitochondria are unable to process the excess of pyruvate, leading to an activation of the alternative anaerobic metabolic pathways, resulting in excessive levels of lactate [8]. Type A is associated with hypoperfusion and hypoxia, leading to oxygen consumption/delivery mismatch and resulting in anaerobic glycolysis. Pathophysiology of type B, on the other hand, is not associated with tissue hypoxia. Know causes of type B lactic acidosis include impaired pyruvate dehydrogenase activity (thiamine deficiency), impaired production by neoplastic cells, drug-induced mitochondrial lactate to glucose (nucleoside reverse transcriptase inhibitors, linezolid, propofol, metformin, theophylline, epinephrine, salicylates), and impaired lactate to glucose (hepatic dysfunction) [9].

Lactic acidosis and hyperlactacidemia are common metabolic disturbances in critically ill patients. In asthma exacerbations, lactic acidosis may result from hypoxemia, hypoperfusion, overproduction due to increased work of the respiratory muscles, and drugs such as β2-agonists [10]. There are no diagnostic criteria for β2-agonist-induced lactic acidosis, and therefore thorough clinical evaluation and careful assessment of alternative causes are essential [11]. In this case, the patient was not in shock, had adequate oxygen levels, had no systemic or regional hypoperfusion, and liver dysfunction and diabetic ketoacidosis were ruled out. Other pharmacological agents besides salbutamol were also excluded.

In a recent prospective study by Ruman-Colombier et al. on children admitted with acute, moderate, and severe asthma, 26% presented with lactic acidosis (lactate > 5 mmol/l and AG ≥ 16 mmol/l), and nebulized salbutamol was a risk factor [12]. Furthermore, Rodrigo et al. showed that high lactate concentrations can develop during the first hours of inhaled β2-agonist treatment in adult patients with severe acute asthma treated with high doses of albuterol [7]. In a systematic review, the most frequently identified agents associated with hyperlactatemia were epinephrine and albuterol. Drug-induced hyperlactatemia was reported in 31.1% with a median time of lactate clearance of 1.9 days [13].

In this case, the patient became tachypnoeic, which could easily mimic an acute deterioration of asthma and instigate further treatment with β2-agonist. Clinical assessment can be challenging and requires careful physical and arterial blood gas analysis examination. Hypokalaemia, in addition to metabolic acidosis and hyperlactacidemia, may increase the suspicion of a SABA adverse effect. In specific situations, using spirometry to measure peak flow can help in distinguishing between salbutamol-induced lactic acidosis (improvement of peak flow) and lactic acidosis secondary to hypoxemia/acute deterioration (decrease in peak flow rate) [1].

Management of patients with lactic acidosis induced by SABA is supportive and requires drug withdrawal. In previous reports, timely recognition and eviction resulted in an overall good prognosis with full recovery [10]. Our patient’s lactate levels increased a few hours after salbutamol administration, rapidly achieving very high levels and returning to normal within 12 hours of drug discontinuation (Table 1). A SABA-related lactic acidosis should be suspected whenever an isolated increase in serum lactate is accompanied by significant clinical improvement.

In such circumstances, the most appropriate treatment is SABA eviction while continuing other recommended combination therapies, such as ipratropium bromide, oral/intravenous corticosteroids, and magnesium sulfate. Should invasive mechanical ventilation be necessary and the condition remains refractory to standard medical therapy, other options, including helium-oxygen therapy or ketamine, may be available, although the evidence supporting their use is weak [14,2].

## Conclusions

Short-acting β2 agonists, such as salbutamol, remain the first line in the treatment of acute asthma due to their ability to provide rapid symptomatic relief. Studies have demonstrated greater efficacy of nebulized versus intravenous salbutamol, with fewer associated adverse effects. There are previously reported cases of severe lactic acidosis secondary to intravenous salbutamol. Although it is most often associated with its intravenous administration, we report this rare adverse effect of salbutamol given through continuous nebulization and the subcutaneous route. The development of otherwise unexplained lactic acidosis in such patients should alert physicians to the possibility of a β2 agonist side effect.
